# Pharmacological inhibition of p38 MAPK reduces tumor growth in patient-derived xenografts from colon tumors

**DOI:** 10.18632/oncotarget.3816

**Published:** 2015-04-14

**Authors:** Jalaj Gupta, Ana Igea, Marilena Papaioannou, Pedro Pablo Lopez-Casas, Elisabet Llonch, Manuel Hidalgo, Vassilis G. Gorgoulis, Angel R. Nebreda

**Affiliations:** ^1^ Institute for Research in Biomedicine (IRB Barcelona), Barcelona, Spain; ^2^ Department of Histology and Embryology, School of Medicine, University of Athens, Athens, Greece; ^3^ Spanish National Cancer Research Centre (CNIO), Madrid, Spain; ^4^ Biomedical Research Foundation of the Academy of Athens, Athens, Greece; ^5^ Faculty Institute of Cancer Sciences, University of Manchester, Manchester, UK; ^6^ Institució Catalana de Recerca i Estudis Avançats (ICREA), Barcelona, Spain

**Keywords:** colon cancer, p38 MAPK, mouse xenograft, therapy

## Abstract

Colorectal cancer is a major health problem and the second cause of cancer related death in western countries. Signaling pathways that control tissue homeostasis are often deregulated during tumorigenesis and contribute to tumor development. Studies in mouse models have shown that the p38 MAPK pathway regulates homeostasis in colon epithelial cells but also plays an important role in colon tumor maintenance. In this study, we have investigated the role of p38 MAPK signaling in patient-derived xenografts (PDXs) from three different human colon tumors representing clinical heterogeneity and that recapitulate the human tumor conditions both at histological and molecular levels. We have found that PH797804, a chemical inhibitor of p38 MAPK, reduces tumor growth of the three PDXs, which correlates with impaired colon tumor cell proliferation and survival. The inhibition of p38 MAPK in PDXs results in downregulation of the IL-6/STAT3 signaling pathway, which is a key regulator of colon tumorigenesis. Our results show the importance of p38 MAPK in human colon tumor growth using a preclinical model, and support that inhibition of p38 MAPK signaling may have therapeutic interest for colon cancer treatment.

## INTRODUCTION

Colorectal cancer (CRC) is the second cause of cancer related mortality in developed countries. Colorectal tumors are of epithelial origin and develop from sequential mutations in several signaling pathways including Wnt, K-Ras, p53 and transforming growth factor (TGF)-β [1, 2]. At the time of diagnosis, about 35% of patients have stage IV metastatic disease and 20-50% of patients with stage II or III disease will progress to stage IV at some point during the course of the disease [3, 4]. CRC typically metastasizes to liver and lung. Unfortunately, 5-year survival rate for metastatic colon cancer is below 10% [5]. Therefore, it is necessary to further understand the biology of CRC in order to develop effective treatments.

Mouse models have been widely used to mimic human CRC, including expression of APC^min^ for familial adenomatous polyposis (FAP) colon cancer and chemical treatments with azoxymethane (AOM) and dextran sodium sulfate (DSS) for colitis-associated colorectal cancer (CAC). CAC represents 1-2% of worldwide CRC incidence, while FAP cases represent approximately 1% of total CRC diagnoses per year [6, 7]. Perhaps the best model to mimic human sporadic CRC is to use AOM treatment without DSS, as colorectal tumors formed in this case recapitulate key human pathological features of human CRC. However, tumor induction by AOM can easily take more than 6 months with tumor multiplicity and penetrance depending on mouse strain and the AOM batch [8].

While both APC^min^ and AOM/DSS models have greatly enhanced our understanding of the basic biology underlying CRC, these models do not allow accurate testing of potential therapies [9]. Patient-derived xenografts (PDXs) are emerging as reliable models that are closer to clinical settings in different tumor types [10-13]. PDXs can be considered as predictive preclinical models [9] and have provided promising platforms for therapeutic decision-making in patients with solid tumors [14].

The mitogen-activated protein kinase (MAPK) p38α can regulate many processes important for tissue homeostasis and that are often deregulated in cancer [15]. Several studies support a tumor suppressive role for p38α during the onset of malignant transformation [16-18]. Recent work has shown that p38α signaling is important for maintaining the homeostasis of the colon epithelium in mice [19-21]. Moreover, specific downregulation of p38α in intestinal epithelial cells enhances AOM/DSS-induced CAC [20, 22]. However, unlike many tumor suppressors that are inactivated during the malignant transformation process, inactivating mutations for p38α have not been consistently detected in human solid tumors. This probably reflects that tumor cells can benefit from the versatility of this signaling pathway. In line with this idea, p38α signaling has been shown to be important for the survival and proliferation of colon tumor cells *in vitro* and in mouse models [20, 23-26]. These observations make the p38α pathway a potential therapeutic target.

Human tumors are heterogeneous in nature, therefore the response to anti-cancer drugs varies among different human tumors. We have used PDXs from three human colorectal tumors with distinct properties and show that inhibition of p38 MAPK signaling reduces colon tumor growth in all cases.

## RESULTS

### Human colon tumor samples and generation of PDXs

**Figure 1:**
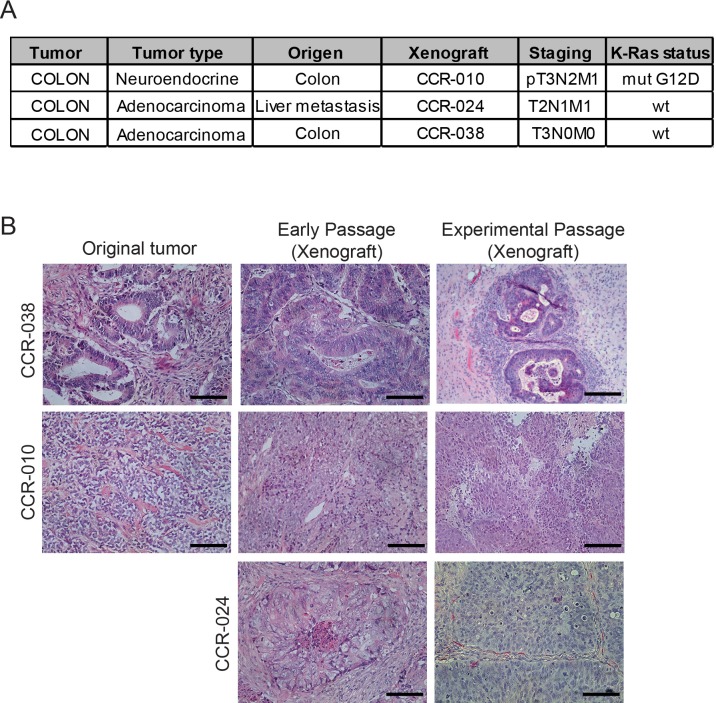
Characteristics of human colon tumors used for xenografts (**A**) clinical characteristics including stage, tumor type and K-Ras mutation status of the three human tumors used to generate PDXs. (**B**) representative H&E stained sections of the original tumors and of xenografts both at an early passage (Px2 in CCR-038 and CCR-010 and Px3 in CCR-024) and the passage used for the experiments (Px3 in CCR-038, Px5 in CCR-010 and Px6 in CCR-024). Scale bars, 100 μm.

To evaluate the role of p38 MAPK signaling in PDXs, we chose three different human colon tumors with distinct properties. Tumor origin, staging and K-Ras mutation status of these tumors are summarized in Figure 1A.

PDXs were generated by direct transplantation of colorectal tumor tissues into female nude mice. Once xenograft models were successfully established, tumors were re-implanted into a panel of female nude mice to expand the colony. To confirm that the PDXs recapitulated the original human colon tumors, we analyzed the histology and the K-Ras mutation status. Histological analysis revealed that both early and experimental passages of the CCR-038 and CCR-010 PDXs were very similar to the corresponding original tumors (Figure 1B). The original human tumor CCR-038 was a moderately differentiated adenocarcinoma while CCR-010 was a neuroendocrine carcinoma. We could not obtain the original sample of the human tumor CCR-024, but this model also retained the histological features of moderate to poor differentiation in the early and experimental passages (Figure 1B).

To further examine potential histological differences, the CCR-010 original human tumor and the PDXs were immunostained for CD56, a known marker for neuroendocrine differentiation. We found no differences in CD56 expression between the original tumor and the PDXs (Suppl. Figure S1). Similarly, PAS staining was used in model CCR-038 to detect mucin-secreting cells, which indicate adenocarcinomas. Again, no differences were found between the CCR-038 original tumor and the PDXs (Suppl. Figure S1), suggesting that cellular differentiation was not significantly altered in the tumors of the PDXs. Moreover, K-Ras mutation status was also confirmed in the experimental PDXs compared with the parental human tumors (Suppl. Figure S2). Altogether, these data indicate that histological and genetic characteristics are conserved in different passages of the PDXs and that these models can be used as a tool to recapitulate the human tumor conditions.

### Inhibition of p38 MAPK signaling reduces tumor growth in PDXs

**Figure 2:**
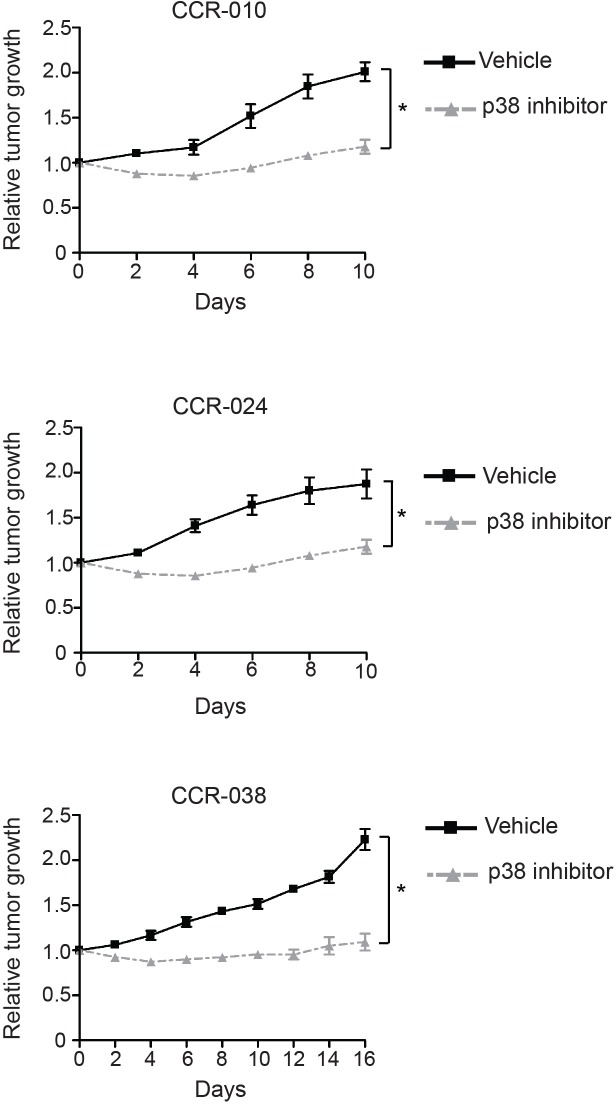
Tumor growth in PDXs treated with vehicle or p38 MAPK inhibitor Mice with xenografted tumors of about 150-200 mm^3^ were treated orally with the p38 MAPK inhibitor PH797804 or vehicle and tumor growth was monitored. CCR-10 and CCR-024 were treated for 10 days and CCR-038 for 16 days. Tumor sizes were measured at the indicated time points and normalized to the original size of each tumor when the treatment began. Graphs show the relative tumor growth in vehicle or PH797804-treated PDXs. Data represent means ± SEM (n ≥ 4). ^*^, *p* < 0.05.

Pharmacological inhibition or genetic downregulation of p38 MAPK signaling in established AOM/DSS-induced colon tumors reduces tumor burden in mice [20]. To investigate the role of p38 MAPK signaling in the PDXs from CRC, we used the inhibitor PH797804. This chemical compound effectively inhibits the p38α and p38β MAPKs, without affecting other MAPKs such as ERK1/2 and JNK, and it is used in clinical trials for inflammatory diseases [27]. Tumors in PDXs were allowed to grow up to a measurable size (150-200 mm^3^) and then mice were randomized into two groups, which received either PH797804 or vehicle. Models CCR-010 and CCR-024 showed a decrease in tumor size when treated with PH797804 during the first 5-7 days. Then, tumors started to grow again although significantly slower than the vehicle treated tumors. These two models were treated for 10 days (Figure 2). Model CCR-038 showed a more pronounced growth inhibition during all the treatment with PH797804. Due to the different response observed, in this case we extended the treatment until day 16 to confirm that tumor growth inhibition was maintained (Figure 2). Therefore, tumor growth was significantly reduced in the PH797804-treated mice for the three PDX models of CRC, although there were slight differences in the response of each model. These results suggest that p38 MAPK signaling is important for human colon tumor growth.

### Histological analysis of PDXs treated with p38 MAPK inhibitor

**Figure 3:**
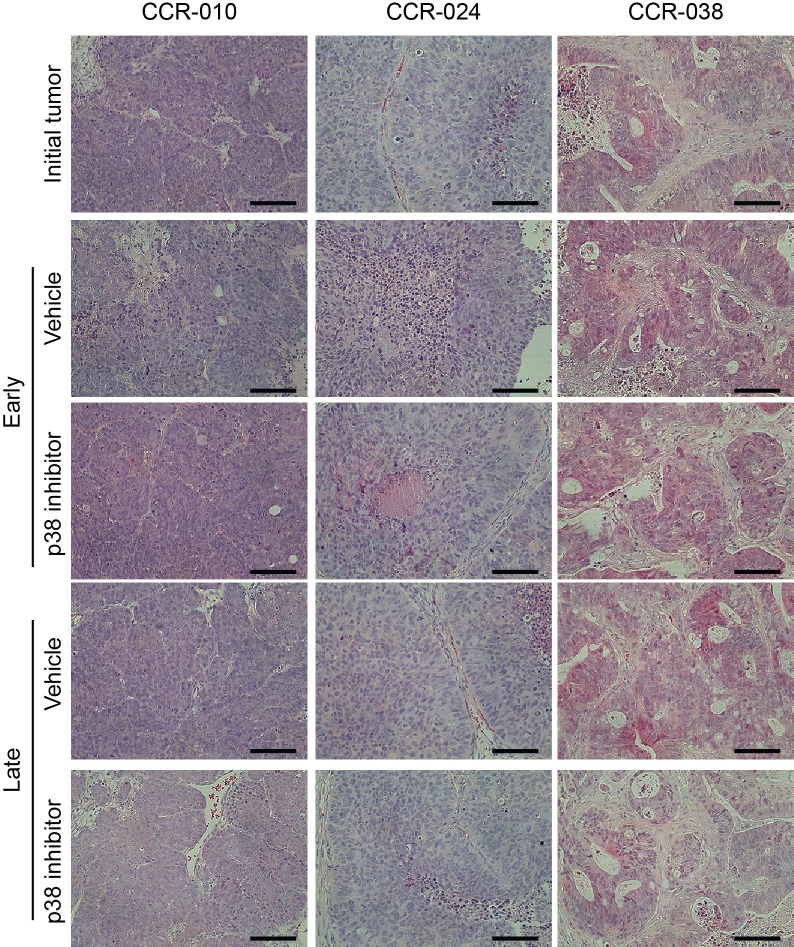
Tumor histology in PDXs treated with vehicle or p38 MAPK inhibitor Representative H&E stained sections of PDXs treated with vehicle or PH797804. Initial tumors refers to the beginning of the treatment, Vehicle early and p38 inhibitor early refer to day 5 of treatment in the case of CCR-010 and CCR-024 and day 8 in the case of CCR-038. Vehicle late and p38 inhibitor late refer to the end of the treatment, day 10 for CCR-10 and CCR-024 and day 16 for CCR-038. Treatment with the p38 MAPK inhibitor did not affect the architecture of tumors compared to vehicle treatment. Scale bars, 100 μm.

To investigate in more detail the reduction in tumor growth observed upon p38 MAPK inhibition, we examined possible alterations in tumor histology. H&E-stained sections from the initial tumors and from PDXs of mice treated with vehicle or the p38 MAPK inhibitor were analyzed at different time points but no differences were found (Figure 3). We also analyzed the presence of mucus secreting epithelial cells by PAS staining as well as the neuroendocrine differentiation by CD56 immunostaining. However, pharmacological inhibition of p38 MAPK affected neither the neuroendocrine differentiation in model CCR-010 nor the mucus secreting epithelial cells in model CCR-038 (Suppl. Figure S1). These results indicate that the reduced tumor growth observed upon p38 MAPK inhibition does not correlate with changes in the differentiation stage of the tumoral cells.

### Cell survival and proliferation in PDXs treated with p38 MAPK inhibitor

**Figure 4:**
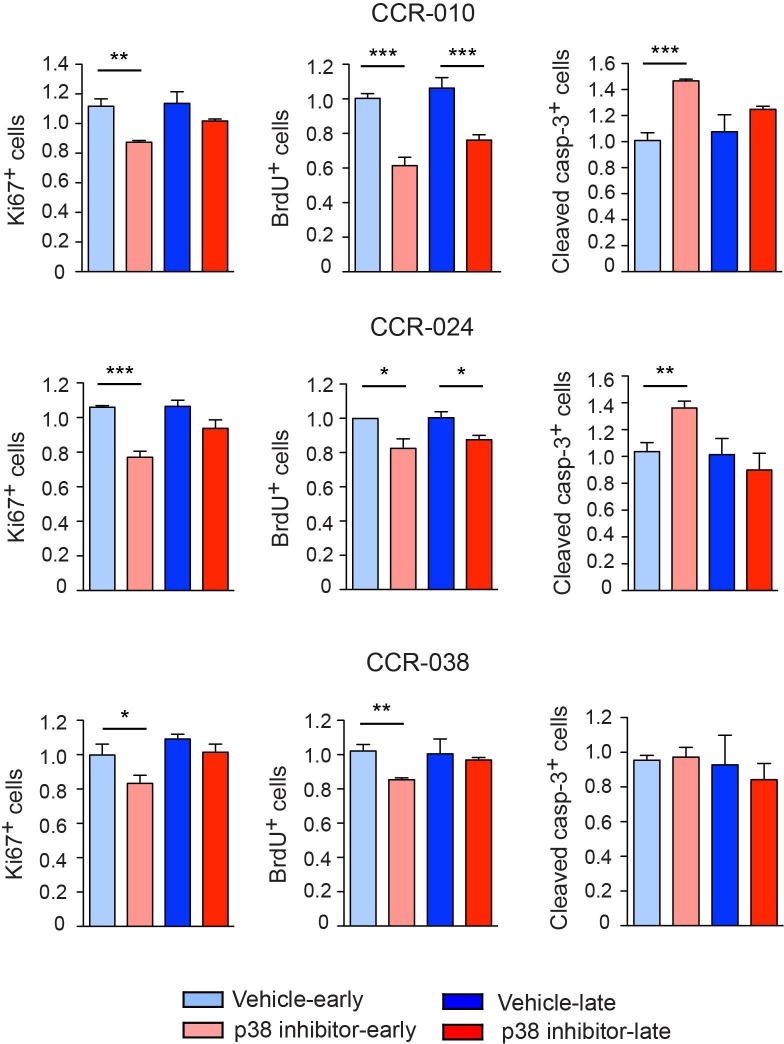
Proliferation and apoptosis in PDXs treated with vehicle or p38 MAPK inhibitor Proliferation and apoptosis levels were determined at early time points (day 5 for CCR-010 and CCR-024 and day 8 for CCR-038) and at the end of the experiment (day 10 for CCR-010 and CCR-024 and day 16 for CCR-038) by counting either Ki67^+^ and BrdU^+^ cells or Cleaved caspase-3^+^ cells. Proliferation and apoptosis in the initial tumors were given the value of 1 and relative indexes were determined for vehicle or PH797804-treated PDXs. At least eight fields were analyzed per tumor. Data represent means ± SEM (n ≥ 4). ^*^, *p* < 0.05; ^**^, *p* < 0.01; ^***^, *p* < 0.001.

Interfering with p38 MAPK signaling in AOM/DSS-induced colon tumors reduces proliferation and enhances death of the tumor cells [20]. Since p38 MAPK inhibition does not affect the differentiation status of the three PDXs tested, we determined cell proliferation and cell death indexes. We analyzed an early time point in the middle of the treatment (day 5 for CCR-010 and CCR-024; day 8 for CCR-038) and a late one at the end (day 10 for CCR-010 and CCR-024; day 16 for CCR-038). We found that for CCR-010 and CCR-024 tumors, cell proliferation measured by counting Ki67^+^ or BrdU^+^ cells was significantly reduced and apoptosis was increased in the mice treated with PH797804 (Figure 4). As expected from the tumor growth curves, the effect of p38 MAPK inhibition in both models was more important at the early times. In fact, impaired growth of CCR-010 and CCR-024 tumors was already observed as early as 2 days after starting the treatment with PH797804 (Figure 2). At late time points, tumors still showed decreased proliferation but apoptosis was not affected (Figure 4), which probably contributed to the PH797804-treated tumors starting to grow again slowly (Figure 2). In contrast, p38 MAPK inhibition reduced cell proliferation at the early time point without affecting apoptosis in CCR-038 tumors (Figure 4). In conclusion, p38 MAPK signaling can regulate the proliferation and survival of colon tumor cells in a tumor-type dependent manner but pharmacological inhibition of this pathway leads to reduced tumor growth in the three PDXs analyzed.

### Effect of p38 MAPK inhibition in signaling pathways of human colon tumor cells

**Figure 5:**
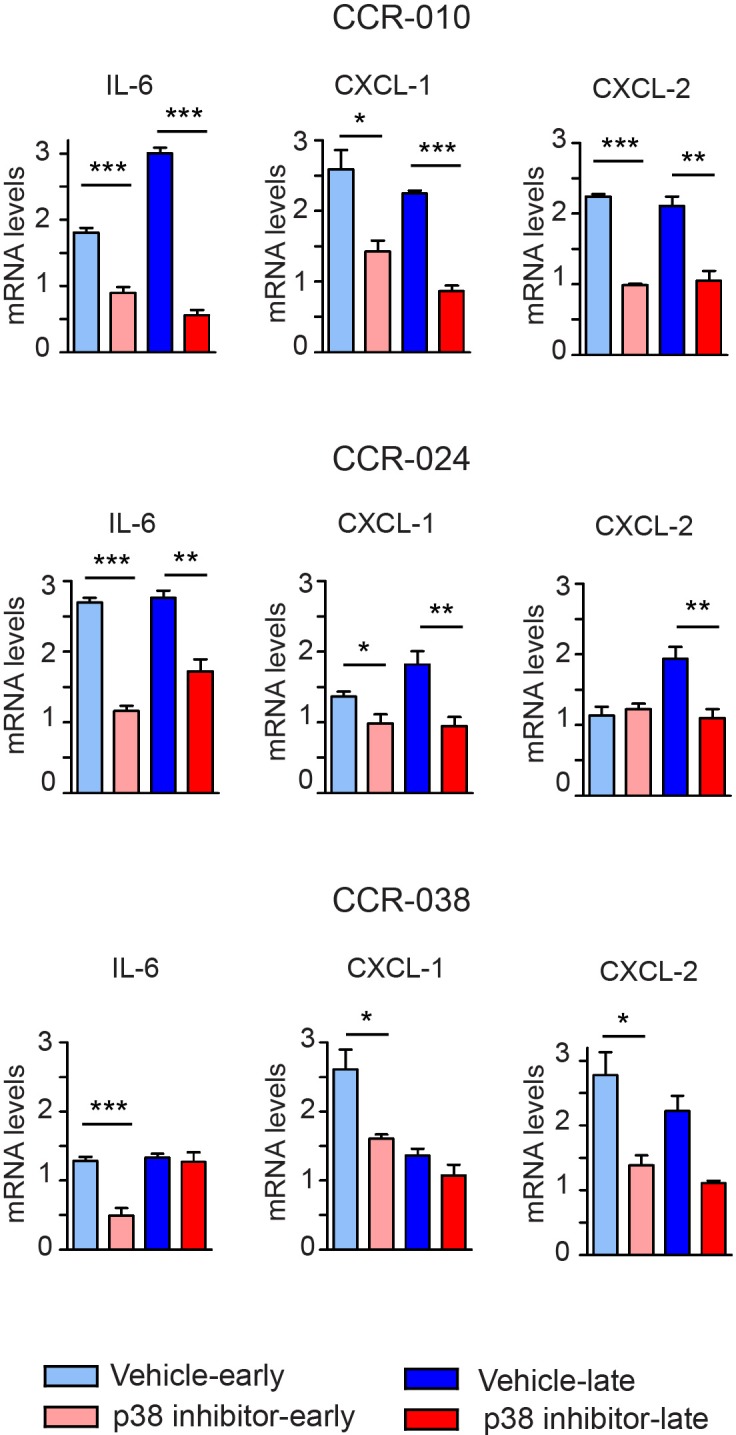
Effect of the p38 MAPK inhibitor in cytokine production by PDXs Relative expression levels of the indicated mRNAs were determined by qRT-PCR and were normalized to the expression levels of initial tumors, which were given the value of 1. Samples of PDXs were collected as in Figure 3. Data represent means ± SEM (n ≥ 4). ^*^, *p* < 0.05; ^**^, *p* < 0.01; ^***^, p < 0.001.

There is good evidence that IL-6 family cytokines (IL-11 and IL-6) and STAT3 signaling are important for the survival and proliferation of colon tumor cells in mouse models [28-32]. Moreover, the chemokine receptor CXCR-2 and its ligands CXCL-1 and CXCL-2 have been proposed to facilitate the growth of spontaneous and inflammation-associated colon tumors [33]. Genetic downregulation of p38α in AOM/DSS-induced mouse colon tumors reduces tumor growth, which correlates with reduced levels of IL-6, IL-11, CXCL-1 and CXCL-2 [20]. Since p38 MAPK inhibition reduced tumor growth in the three PDXs models from CRC, we investigated whether similar mechanisms were involved as in mouse colon tumors. Gene expression analysis showed that in CCR-010 tumors, IL-6, CXCL-1 and CXCL-2 mRNAs were all downregulated at both early and late time points upon p38 MAPK inhibition compared to vehicle treatment (Figure 5). Expression of IL-6 and CXCL-1 mRNAs was also reduced at both time points analyzed in CCR-024 tumors treated with PH797804, while CXCL-2 mRNA expression was only reduced at the late time point (Figure 5). In CCR-038 tumors, the expression of IL-6, CXCL-1 and CXCL-2 mRNAs was significantly downregulated only at the early time point upon PH797804 treatment (Figure 5). In contrast, the expression of IL-11 mRNA was only affected by p38 MAPK inhibition at the late time point in CCR-038 tumors but not in the other two PDXs (Suppl. Figure S3).

Treatment with SB202190, another pharmacological inhibitor of p38α and p38β MAPKs, has been reported to reduce the proliferation and survival of human colon cancer cell lines [23, 25]. This effect of SB202190 correlated with upregulation of the receptor tyrosine kinase HER-3, which was proposed to induce activation of the ERK1/2 pathway, and GABARAP, a protein associated with autophagic vacuole formation [23, 25]. However, the reduced tumor growth observed in PDXs from CRC upon p38 MAPK inhibition with PH797804 did not correlate with consistent changes in the expression of HER-3 or GABARAP mRNAs. In fact, only CCR-038 tumors treated with PH797804 showed some increase in HER-3 expression at the early time point (Suppl. Figure S3). To address this apparent discrepancy, we treated three human colon cancer lines with SB202190 or PH797804. In agreement with previous reports [25], we observed that SB202190 induced autophagic vacuoles in the three colon cancer cell lines. In contrast, PH797804 did not induce the formation of vacuoles in any of the colon cancer cell lines (Suppl. Figure S4A). As a control, we confirmed that SB202190 and PH797804 both efficiently inhibited the UV-induced activation of the p38 MAPK pathway, as determined by the impaired phosphorylation of the downstream targets MK-2 and Hsp27 (Suppl. Figure S4B). Moreover, we found neither upregulation of HER-3 and GABARAP mRNAs nor activation of the ERK1/2 pathway in the colon cancer cell lines treated with either SB202190 or PH79704 compared to the vehicle (DMSO)-treated cells (Suppl. Figures S5A and S5B). Taking together, our results indicate that p38 MAPK inhibition does not affect the expression of HER-3 and GABARAP, which are therefore unlikely to contribute to the reduced tumor growth observed in the PDXs from human colon tumors.

**Figure 6:**
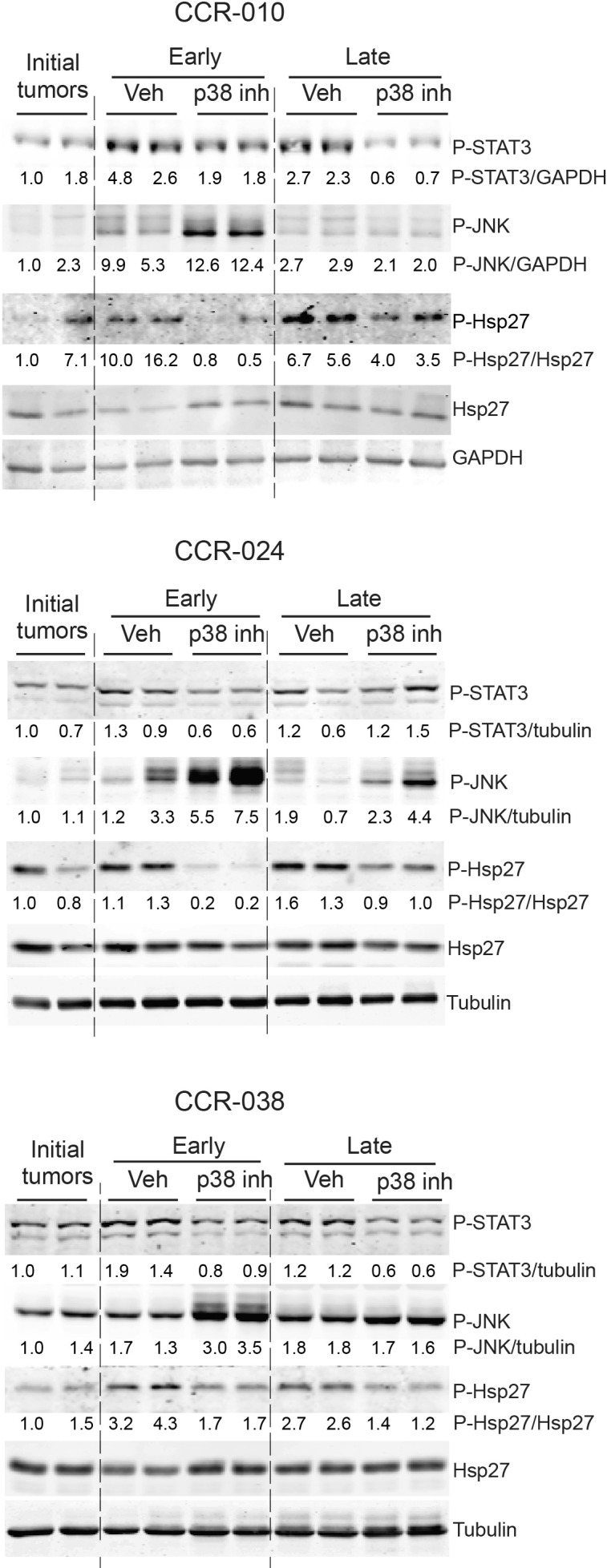
Activation of selected signaling pathways in PDXs treated with p38 MAPK inhibitor Tumor lysates were prepared from PDXs treated with either vehicle or PH797804 and were analyzed by immunoblotting (one tumor per lane) with the indicated antibodies. Samples of PDXs were collected as in Figure 3. Quantifications were performed using ImageJ software and normalized to the loading control indicated in the figure. Indicated values are relative to the initial tumor that was given the value of 1.

Interestingly, the reduced levels of IL-6 mRNA observed in the PDXs upon p38 MAPK inhibition correlated with reduced phosphorylation of STAT3, an important regulator of colon tumor cell proliferation and survival (Figure 6). We also found increased activating phosphorylation of JNK upon p38 MAPK inhibition in the PDXs (Figure 6), which is consistent with the ability of p38 MAPK to negatively regulate JNK signaling and the implication of sustained JNK activation in apoptosis [15, 34]. Inhibition of the p38 MAPK pathway in PH797804-treated PDXs was confirmed by the reduced phosphorylation of the downstream target Hsp27 (Figure 6). We also detected reduced p38 MAPK phosphorylation in PH797804-treated tumors (Suppl. Figure S6). Moreover, increased cell death in PDXs treated with PH797804 correlated with increased expression of the pro-apoptotic protein Bax in CCR-010 (Suppl. Figure S7), and with reduced expression of the anti-apoptotic protein Mcl-1 in CCR-024 (Suppl. Figure S7), both at early time points upon p38 MAPK inhibition. The expression level of Bak did not change in any of the PDXs (Suppl. Figure S7).

Collectively, our data using PDXs that mimic human pathological conditions of CRC based on key histological features and K-Ras mutation status, indicate that p38 MAPK signaling contributes to human colon tumor growth.

## DISCUSSION

Mouse models with genetically or chemically-induced colon tumors have greatly enriched our knowledge of the basic biology of CRC but these models usually lack the complexity of the human tumors. Moreover, murine tumors might not truly represent the therapeutic response of human tumors. To complement the studies with mouse models, assays based on human cancer cell lines have been used for decades as preclinical models for the screening of novel cancer therapeutics. However, human cancer cell lines cultured *in vitro* or grown in mice as xenografts have also limitations, including reduced intra-tumoral heterogeneity, modest diversity of molecular subtypes and lack of human stromal cells [35]. To overcome these limitations, attempts have been made to establish PDXs from several types of tumors [10-13, 35]. Since PDXs are derived directly from patient tumor samples with minimal *in vitro* manipulation, they are expected to provide a more accurate depiction of human tumors. In agreement with previous reports [11, 35], we have confirmed that PDXs from CRC retain key histopathological and K-Ras mutational status of human tumors, suggesting they are histologically and genetically stable in mice and thus serve as a model system for testing new therapeutics. Indeed, the effect of drugs on PDXs from colorectal and pancreatic tumors has been reported to correlate notably with clinical outcome, both in terms of drug resistance and sensitivity [11, 13]. Collectively, PDXs seem closer to the clinical setting and therefore we have used them to investigate the effect of p38 MAPK inhibition on the growth of human colon tumors.

There is good evidence that p38α can function as a tumor suppressor in several mouse models of cancer [17, 18, 20, 22, 36]. It should be noted that all these studies have been performed by genetic deletion of p38α before the tumors are induced, which is not the usual scenario in clinical practice. Interestingly, genetic deletion or pharmacological inhibition of p38α have revealed pro-tumorigenic roles of this signaling pathway in breast and colon cancer models [20, 23, 37, 38] therefore supporting the use of p38 MAPK inhibitors for the treatment of these tumors. In the case of colon cancer, the studies were done using chemically-induced mouse tumors or human cell lines subcutaneously implanted into nude mice, which may not truly reflect the human tumor conditions as discussed above. Thus additional studies using a closer setting to the clinical situation were needed to confirm the role of p38 MAPK signaling in colon tumor progression.

We have used three PDXs models from CRC with different characteristics and have found that tumor growth is substantially retarded upon p38 MAPK inhibition in the three cases. K-Ras mutation plays a significant role in the prognosis of patients with advanced CRC and can affect the response to Cetuximab, a monoclonal antibody-based therapy that improves overall and progression-free survival in CRC patients who do not respond to chemotherapy [39]. Our results show that inhibition of p38 MAPK signaling impairs tumor growth irrespective of K-Ras mutation status or type of colon tumor. It would be interesting to extend our observations using other p38 MAPK inhibitors currently available for clinical trials. However, there seem to be differences in the underlying basis for the reduced tumor growth observed in the PDXs, as p38 MAPK inhibition mainly affects cell proliferation but not survival in CCR-038 tumors, whereas both cell proliferation and survival are impaired in CCR-010 and CCR-024 tumors. Intriguingly, PH797804 appeared to be more effective inhibiting the growth of CCR-038 tumors, although the inhibition of cell proliferation was similar in the three PDXs. This could be related to the slower growth rate of CCR-038 compared to the other two models.

At the molecular level, impaired tumor growth in the three PDXs treated with p38 MAPK inhibitor correlates with downregulation of the chemokines CXCL-1 and CXCL-2 and the cytokine IL-6, which are all known to play key roles in colon tumorigenesis [29, 33, 40]. These effects could be mediated by the regulation of IL-6 and CXCL1/2 mRNA stability by p38α [41]. Regulation of the IL-6/STAT3 pathway by p38α impinges upon tumor cell proliferation and survival in mouse models of colon cancer [20]. Our results extend this observation and suggest an important role for p38α in the regulation of IL-6/STAT3 signaling in human colon tumors. In contrast, IL-11 can also induce STAT3 activation [42], but it does not seem to be affected by p38 MAPK inhibition in these models. Only PH797804-treated CCR-038 tumors show a small increase in IL-11 mRNA levels, which does not correlate with changes in STAT3 phosphorylation, cell proliferation or apoptosis, suggesting that IL-11 is not produced at high enough levels to engage STAT3 activation. Inhibition of p38 MAPK in the PDXs also results in increased JNK activation, which is consistent with the implication of sustained JNK activation in apoptosis [15, 34]. However, we could detect no changes in the expression of the JNK target genes c-JUN, p16INK4a and BIM between vehicle and PH797804-treated PDXs (data not shown), suggesting that the observed JNK activation does not result in general gene expression changes. Expression analysis of Bcl-2 family proteins suggests that the mechanism by which p38α regulates tumor cell survival, probably varies depending on the colon tumor type.

The p38 MAPK inhibitor SB202190 has been reported to impair the proliferation of human colon cancer cell lines *in vitro* and in mouse xenografts, which has been correlated with autophagy induction and increased expression of HER-3 and GABARAP [23, 25]. However, in agreement with other studies [43], our results indicate that the induction of autophagic vacuoles is a p38 MAPK-independent effect of SB202190. Moreover, neither GABARAP nor HER-3 mRNAs were consistently upregulated upon p38 MAPK inhibition in the PDXs and our studies with cancer cell lines suggest that long-term incubation with DMSO or high cell confluency may induce HER-3 and GABARAP independently of p38 MAPK signaling.

Taken together, our studies with PDXs from CRC extend previous reports using human colon cancer cell lines or AOM/DSS-induced mouse colon tumors, and support the potential interest of using p38 MAPK inhibitors for CRC treatment. Along this line, enhanced levels of phosphorylated p38 MAPK have been detected in human colon tumors [44] and a recent report that analyzed by immunohistochemical staining the p38 MAPK activation status in 316 CRC patients, concluded that high phospho-p38 MAPK levels predict worse prognosis for CRC [45]. The inhibition of p38 MAPK signaling has been also reported to sensitize human colon and breast cancer cells to apoptosis induced by the chemotherapeutic drugs such as cisplatin and FOLFIRI [26, 38, 44, 46]. These studies are encouraging but are mainly based on the use of established cell lines *in vitro* or in xenografts. Therefore, p38 MAPK inhibitors combined with chemotherapeutic drugs or other therapies should be rigorously tested for accuracy and reproducibility in PDXs from several patients, ideally using a panel of human tumors that represent the heterogeneity observed in the clinic.

## MATERIALS AND METHODS

### Generation of PDXs from colorectal tumors

PDXs were generated as described previously [14]. Briefly, tumor tissue specimens were cut into 2- to 3-mm^3^ pieces in antibiotic-containing RPMI medium. Non-necrotic tumor pieces were selected and immersed in Matrigel (BD Biosciences #354234). Under anesthesia with isoflurane, one tumor piece was implanted subcutaneously by a small incision in each side of the lower back into 5-6 weeks old female athymic Nude-Foxn1^nu^ mice (Harlan Laboratories). Tumors were harvested when they reached a size of 1500 mm^3^ (Px1 xenografts). Xenografts from Px1 mice were divided into small pieces (approx. 3 mm^3^) and then implanted again subcutaneously as described above to obtain Px2 xenografts. This process was further repeated and the experiments were performed on xenografts between Px3 and Px6. For the experiments, xenografts were allowed to grow until they reached a size of 150-200 mm^3^ and then mice were randomized into two groups for the treatment as described below. Tumor size was measured twice a week by a digital caliper using the following formula: Tumor volume = (length × width^2^)/2. At the end of the treatment, tumors were harvested and divided in three pieces. One tumor piece was fixed in 10% formalin solution (Sigma, #HT-501128) at RT for 24 h and then was paraffin-embedded. The other two pieces were snap frozen for RNA and protein analysis. Mice were housed according to national and EU regulations and protocols were approved by the animal care and use committee of SEA-PCB. Mice were housed in a temperature-controlled facility using individually ventilated cages, standard diet and a 12-h light/dark cycle. Human material was donated following all the guidelines established in the protocol approved by the IRB of reference (CEIC-Grupo Hospital de Madrid).

### K-Ras mutation analysis

Genomic DNA was isolated from snap frozen tumor tissues using QIAamp DNA Mini Kit (QIAGEN #51304). Samples were diluted to 100 ng/μl and PCR was performed using the following primers for K-Ras: Forward GGCCTGCTGAAAATGACTGA; Reverse GTCCTGCACCAGTAATATGC. PCR products were purified using DNA clean & Concentrator^TM^ kit (ZYMO Research #D4006) and sequenced using forward and reverse primers. Mutations were detected by observing individual chromatograms.

### p38 MAPK inhibition in mice

The inhibitor of p38α and p38β MAPKs PH797804 [27] was obtained from Selleckchem (#S2726) and was dissolved in PBS containing 0.5% Methyl cellulose (Sigma #M7140) and 0.025% Tween 20 (Sigma #P1379) at a concentration of 1 mg/ml. Mice were weighted and a daily dose of 10 mg/kg body weight was administered by oral gavage for 10 consecutive days for the PDX CCR-010 and CCR-024 and 16 consecutive days for CCR-038. Control mice were similarly administered vehicle (PBS with 0.5% Methyl cellulose and 0.025% Tween 20).

### Histochemical staining

Paraffin-embedded tumor sections were stained with hematoxylin and eosin (H&E) and analyzed by pathologists in blinded fashion for tumor grading. Immunostainings were performed using antibodies against CD-56 (Abcam #ab8233; 1:50, overnight 4ºC), BrdU (BD Biosciences #347580; 1:100, 1 h RT), Ki67 (Novocastra, #Ki67P-CE, 1:1000, 1 h RT) and cleaved caspase-3 (Cell Signaling #9661; 1:200, 1 h RT). The secondary antibodies used were HRP conjugated anti-rabbit (ImmunoLogic #DPVR110HRP, 45 min at RT), anti-mouse (Dako #P0447; 1:100, 30 min at RT) and anti-rat (Dako #P0450; 1:75, 30 min at RT). Signals were visualized with DAB (3,3-diaminobenzidine), using hematoxylin as a counterstaining.

Periodic acid-Schiff (PAS) reagent was used to detect mucus-secreting cells. Slides were incubated with an aqueous solution of 1% periodic acid for 10 min at RT, followed by incubation with Schiff's reagent (Merck, HX383284) for 20 min. Hematoxylin staining was used as a counterstain.

### Proliferation and apoptosis analysis

For proliferation, BrdU (Roche #10280879001) was intraperitoneally injected (1 mg /10 g body weight) and 2 h later the mice were sacrificed and sections were stained with BrdU antibody. Consecutive sections were also stained with the Ki67 antibody to confirm the proliferation index. For apoptosis, sections were stained with antibody for cleaved caspase 3. Proliferation and apoptosis levels were determined by counting the number of positive cells and the total number of cells in at least eight random fields at 20x magnification.

### Cell culture

Human colon cancer cell lines HT-29, Caco-2 and DLD-1 were provided by Gavin Whissell and Eduard Batlle (Colorectal cancer laboratory, IRB Barcelona) and were expanded, tested for mycoplasma contamination and then frozen in vials that were directly used for experiments. Cells were grown in Dulbecco's modified Eagle's medium supplemented with 10% heat-inactivated fetal bovine serum, 1% l-glutamine and 1% penicillin-streptomycin. The p38α and p38β MAPK inhibitors SB202190 (10 μM) and PH797804 (2 μM) were used to treat cells. To induce p38 MAPK activation, cells were UV-treated (60 J/m^2^) and collected 45 min later.

### Immunoblotting

Tumor pieces or cultured cells were lysed in buffer containing 1% NP40, 150 mM NaCl, 50 mM Tris HCl pH 7.5, 2 mM EDTA, 2 mM EGTA, 20 mM sodium fluoride, 2 mM PMSF, 2 μM microcystin, 2 mM sodium orthovanadate, 1 mM DTT and 1x EDTA-free complete protease inhibitor cocktail (Roche, #11873580001), using Precellys homogenization and lysis instrument (Bertin technologies). Protein content was quantified using the Bradford assay with BSA as standard (Bio-Rad), and 40 μg of total protein lysate were separated on SDS-PAGE and transferred to nitrocellulose membrane (Whatman #10401396). After blocking (5% non-fat milk and 1% BSA in PBS, 1 h at RT) membranes were incubated at 4ºC overnight with the following primary antibodies: p38α (#9218; 1:1000), phospho-p38 MAPK (#9211; 1:1000), phospho-STAT3 Tyr705 (#9145; 1:600), phospho-ERK1/2 (#9191, 1:1000), phospho-MEK (#9154, 1:500), phospho-Hsp27 Ser82 (#2401; 1:700), phospho-Hsp27 Ser78 (#2405, 1:1000), phospho-MK2 (#3007, 1:500), Mcl-1(#5453; 1:600), Bak (#3814; 1:1000), Bax (#2772; 1:1000) from Cell Signaling; phospho-JNK (#C12541; 1:500) from BD; Hsp27 (#SC-1049; 1:700) from Santa Cruz. Tubulin (Sigma #T9026) or GAPDH (Sigma #G8795) were used as loading controls. After three washes with PBS, membranes were incubated with Alexa Fluor 680 or 800-conjugated secondary antibodies (Invitrogen; 1:5000) for 1 h at RT and were visualized using Odyssey Infrared Imaging System (Li-Cor, Biosciences).

### RNA extraction and qRT-PCR

Total RNA was extracted from tumor pieces or cultured cells using TRIzol (Invitrogen) or PureLink RNA mini kit (Ambion #12183018A). After DNase I treatment (Roche #04716728001), total RNA (1-2 μg) was reverse transcribed using a Super script II Reverse Transcriptase (Invitrogen #18064-014) and Random primers (Invitrogen #48190-011). qRT-PCR was performed in triplicates using 4 μl of 1/12 diluted cDNA and SYBR green (Bio-Rad #1708886) in 20 μl total volume on a Bio-Rad C1000 thermal cycler machine. Relative quantities (Δ cycle threshold values) were obtained by normalizing against GAPDH. The primers used are listed in the Supplemental Table.

### Statistical methods

Data are presented as mean ± SEM. Statistical significance was determined by Student's *t* test or by two-way ANOVA with Bonferroni post-test for PDX tumor growth curves using GraphPad Prism 4 software. p values less than 0.05 were considered statistically significant.

## SUPPLEMENTARY MATERIALS, FIGURES AND TABLE


