# Supplementary material for: Pharmacological inhibition of p38 MAPK reduces tumor growth in patient-derived xenografts from colon tumors
Source: Oncotarget. 2015 Apr 14;6(11):8539–51. doi: 10.18632/oncotarget.3816 (PMC4496165; doi:10.18632/oncotarget.3816)
Supplement: Supplementary file 1 [file oncotarget-06-8539-s001.pdf]

Pharmacological inhibition of p38 MAPK reduces tumor growth in patient-derived xenografts from colon tumors

Supplementary Material

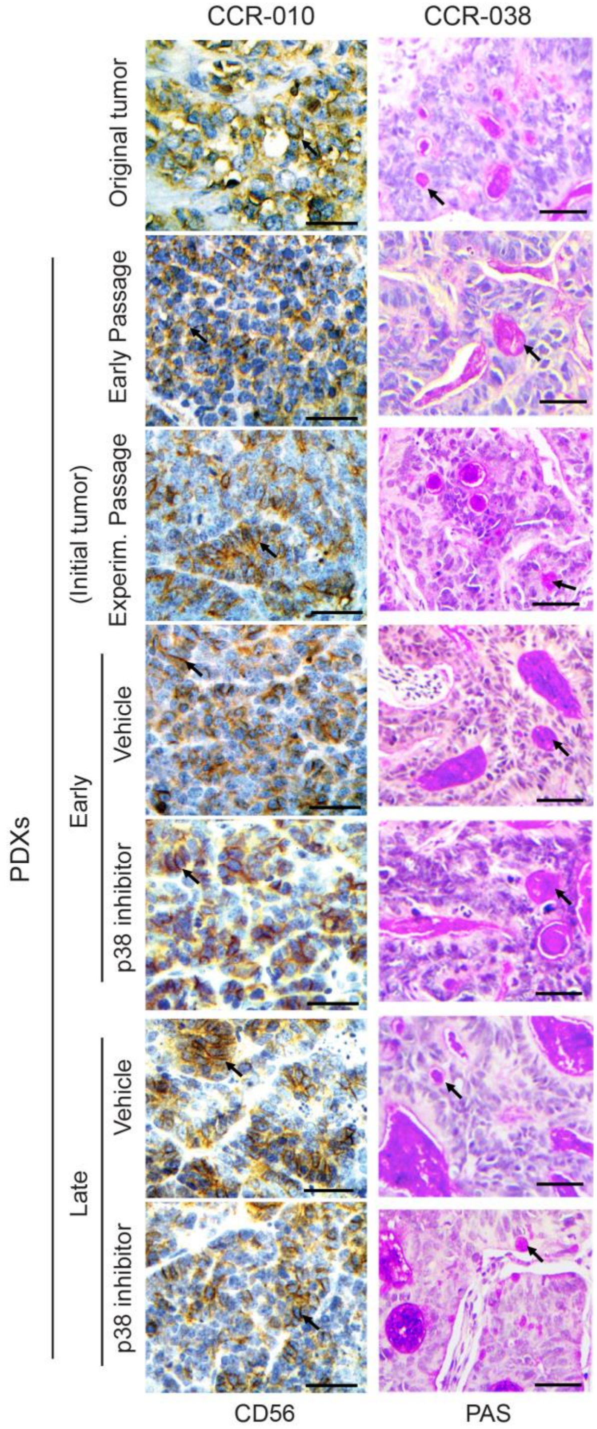

Figure S1: Differentiation markers in human tumors and in PDXs upon p38 MAPK inhibition. Representative staining of original tumors and the xenografted tumors from mice treated with vehicle or PH797804. CCR-010 tumors retain the expression of the CD-56

marker for neuroendocrine differentiation; arrows indicate CD56$^+$ immunostaining. CCR-038 tumors retain PAS staining, a marker of mucus secreted by goblet cells. Arrows denote mucin within neoplastic glands. Inhibition of p38 MAPK has no effect on the expression of these markers in PDXs. Scale bars, 50 μm.

Tumor model	Original tumors	Xenografts (Px2)	Xenografts (Experimental Passage)
**CCR-010**	mut G12D	mut G12D	mut G12D
**CCR-024**	wt	wt	wt
**CCR-038**	wt	wt	wt

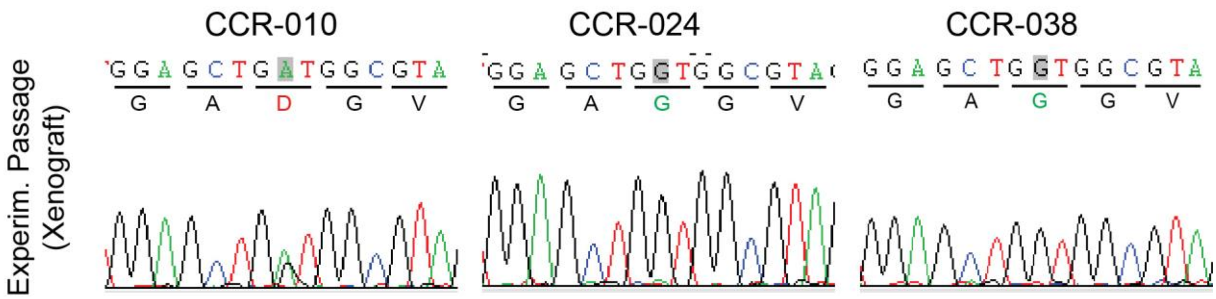

Figure S2: K-Ras status in human colon tumors and corresponding PDXs. Comparison of K-Ras status in the original tumors and in xenografted tumors both at an early passage and the experimental passage. All PDXs have the same K-Ras status as the original human tumors. Electropherograms obtained from the indicated xenografts in experimental passage are shown.

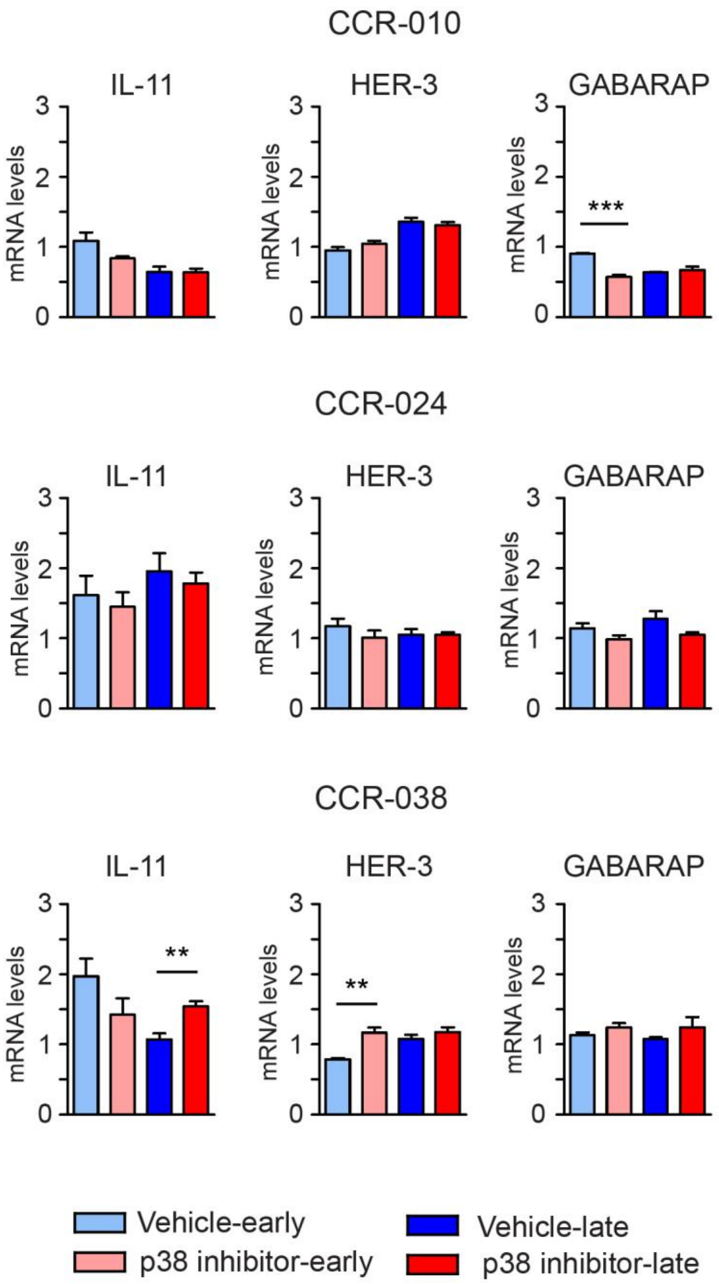

Figure S3: Expression of IL-11, HER-3 and GABARAP in PDXs upon p38 MAPK inhibition. Relative expression levels of the indicated mRNAs in PDXs were determined by qRT-PCR and were normalized to the expression levels of initial tumors, which were given the value of 1. Vehicle early and p38 MAPK inhibitor early refer to day 5 in the case of CCR-010 and CCR-024 and day 8 in the case of CCR-038. Vehicle late and p38 inhibitor late refer to the end of the treatment, day 10 for CCR-10 and CCR-024 and day 16 for CCR-038. Data represent means ± SEM ($n \geq 4$).

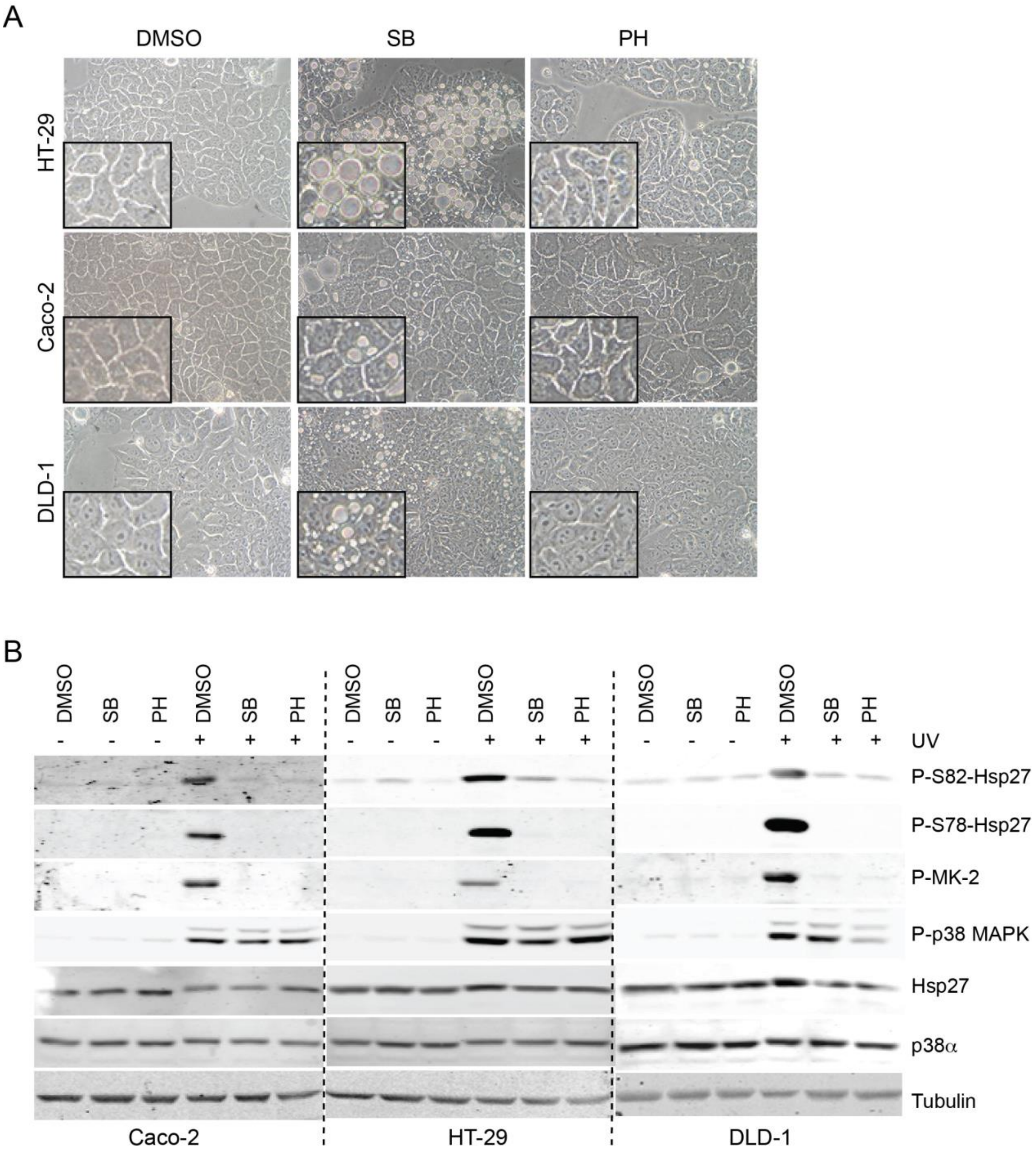

Figure S4: Inhibition of p38 MAPK using PH797804 or SB202190 in human colon cancer cell lines. (**A**) the indicated colon cancer cell lines were incubated with DMSO, SB202019 (10 μM) or PH797804 (2 μM) for 48 h. Cells were photographed with a phase-contrast microscope. SB202019-treated cells form autophagic vacuoles while DMSO or PH797804-treated cells do not. (**B**) colon cancer cell lines were treated as in (**A**) and then exposed or not to UV-irradiation. Cell lysates were prepared and analyzed by immunoblotting with the indicated antibodies.

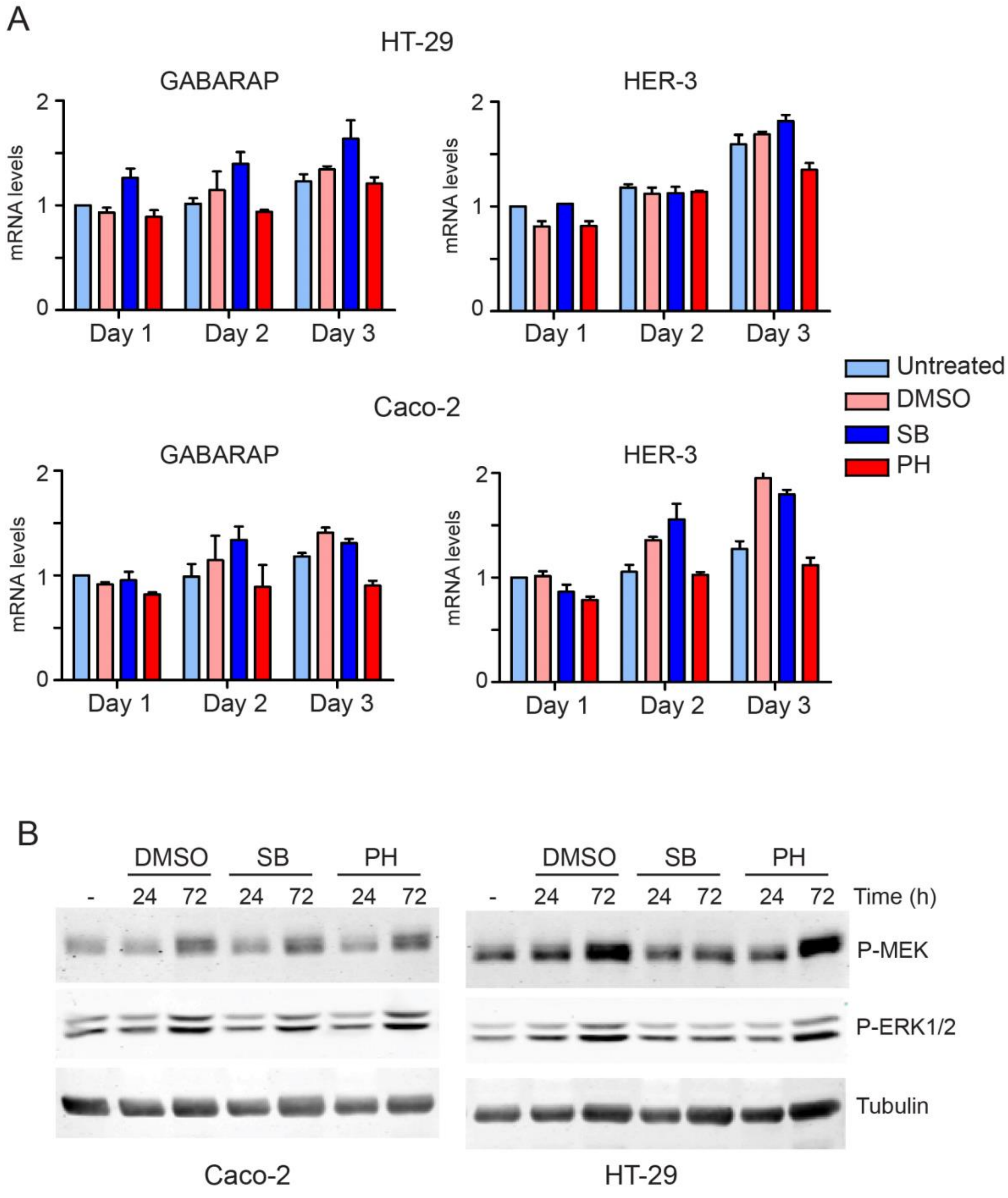

Figure S5: GABARAP and HER-3 expression and activation of the ERK1/2 pathway upon p38 MAPK inhibition in human colon cancer cell lines. (**A**) HT-29 and Caco-2 cells were treated with DMSO, SB202190 (10 µM) or PH797804 (2 µM) for the indicated times. Relative mRNA expression levels were determined by qRT-PCR. Expression levels were normalized to the expression levels of untreated cells that were given the value of 1. (**B**) HT-29 and Caco-2 cells were treated with DMSO, SB202190 or PH797804 for 24 h and 72 h or were left untreated. Cell lysates were prepared and analyzed by immunoblotting with the indicated antibodies.

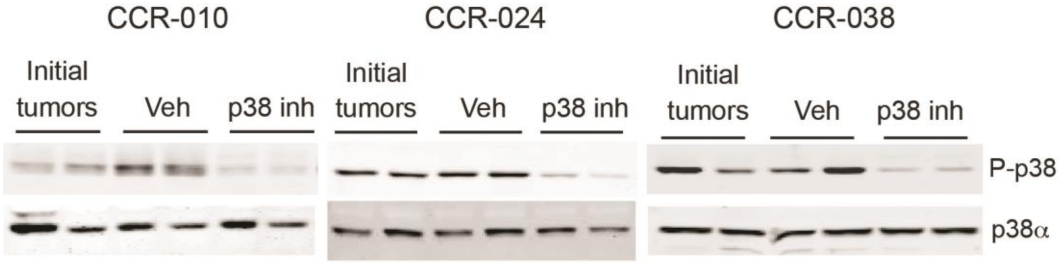

Figure S6: p38 MAPK phosphorylation in PDXs treated with p38 MAPK inhibitor. Tumor lysates were prepared from PDXs treated with either vehicle or PH797804 for 5 days in the case of CCR-010 and CCR-024 or 8 days in the case of CCR-038 and were analyzed by immunoblotting (one tumor per lane) with the indicated antibodies.

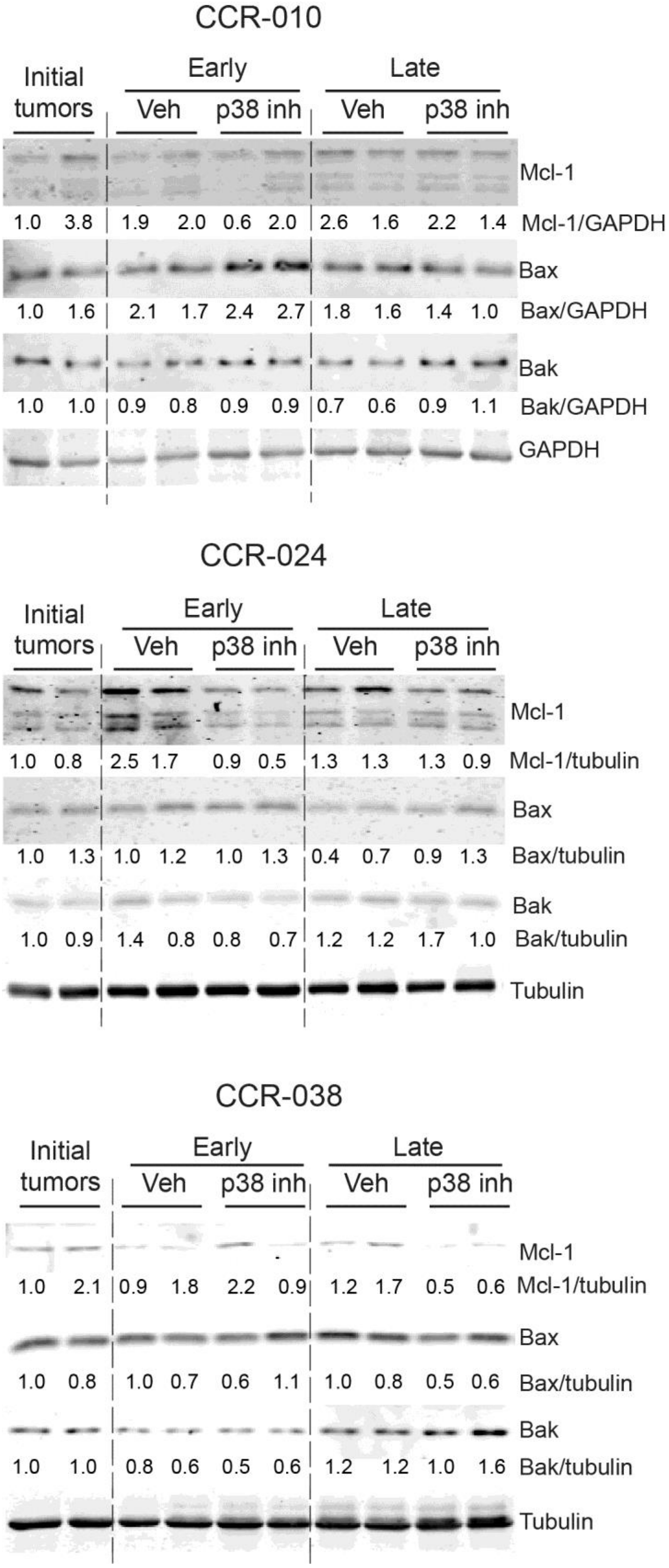

Figure S7: Expression of Bcl2- family proteins in PDXs treated with p38 MAPK inhibitor. Tumor lysates were prepared from PDXs treated with either vehicle or PH797804 and were analyzed by immunoblotting (one tumor per lane) with the indicated antibodies. Initial tumors

refers to the beginning of the treatment, Vehicle early and p38 inhibitor early refer to day 5 in the case of CCR-010 and CCR-024 and day 8 in the case of CCR-038. Vehicle late and p38 MAPK inhibitor late refer to the end of the treatment, day 10 for CCR-10 and CCR-024 and day 16 for CCR-038. Quantifications were performed using ImageJ software and normalized to the loading control indicated in the figure. Indicated values are relative to the initial tumor that was given the value of 1.

Supplemental Table: Primers used for quantitative RT-PCR

**Gene**	**Forward Primer (5'-3')**	**Reverse Primer (5'-3')**
IL-6	CAATCTGGATTCAATGAGGAGAC	CTCTGGCTTGTTCCTCACTACTC
IL-11	CATGAACTGTGTTTGCCGCCT	GGGAATCCAGGTTGTGGTCC
CXCL-1	ATAGCCACACTCAAGAATG	TCTGCAGCTGTGTCTCTCTT
CXCL-2	CGCCCAAACCGAAGTCATAG	AGACAAGCTTTCTGCCCATTCT
HER-3	GGTGATGGGGAACCTTGAGAT	CTGTCACTTCTCGAATCCACTG
GABARAP	CGGGTGCCGGTGATAGTAGA	TGAGATCAGAAGGCACCAGGTA
